# A Self-Diagnosis Method for Detecting UAV Cyber Attacks Based on Analysis of Parameter Changes

**DOI:** 10.3390/s21020509

**Published:** 2021-01-13

**Authors:** Elena Basan, Alexandr Basan, Alexey Nekrasov, Colin Fidge, Ján Gamec, Mária Gamcová

**Affiliations:** 1 Institute for Computer Technologies and Information Security, Southern Federal University, Chekhova 2, 347922 Taganrog, Russia; basanalex@gmail.com (A.B.); alexei-nekrassov@mail.ru (A.N.); 2 Science and Engineering Faculty, Gardens Point Campus, Queensland University of Technology (QUT), Brisbane City QLD 4001, Australia; c.fidge@qut.edu.au; 3 Faculty of Electrical Engineering and Informatics, Technical University of Košice, Letná 9, 042 00 Košice, Slovakia; jan.gamec@tuke.sk (J.G.); maria.gamcova@tuke.sk (M.G.)

**Keywords:** UAV, GPS, cyber threats, anomalies, spoofing, entropy, cyber attacks

## Abstract

We consider how to protect Unmanned Aerial Vehicles (UAVs) from Global Positioning System (GPS) spoofing attacks to provide safe navigation. The Global Navigation Satellite System (GNSS) is widely used for locating drones and is by far the most popular navigation solution. This is because of the simplicity and relatively low cost of this technology, as well as the accuracy of the transmitted coordinates. Nevertheless, there are many security threats to GPS navigation. These are primarily related to the nature of the GPS signal, as an intruder can jam and spoof the GPS signal. We discuss methods of protection against this type of attack and have developed an experimental stand and conducted scenarios of attacks on a drone’s GPS system. Data from the UAV’s flight log were collected and analyzed in order to see the attack’s impact on sensor readings. From this we identify a new method for detecting UAV anomalies by analyzing changes in internal parameters of the UAV. This self-diagnosis method allows a UAV to independently assess the presence of changes in its own subsystems indicative of cyber attacks.

## 1. Introduction

A drone or Unmanned Aerial Vehicle (UAV) is a cyber-physical system combining physical processes, computation, and networking. If an attack on the Global Positioning System (GPS) is executed well, it can lead to serious consequences for a single UAV or a UAV group [1]. In addition to the natural errors and inaccuracies in sensor readings, GPS vulnerabilities also affect the flight controller. These vulnerabilities include the possibility of signal jamming or a signal distortion that disrupts the availability of the GPS signal. GPS vulnerabilities go beyond the natural properties of the transmitted signal. In GPS spoofing attacks, satellites transmitting a GPS signal are tampered with to manipulate the UAV’s navigation system by transmission of fake coordinates [2,3]. Although the GPS signal’s waveform can be implemented in a secure design, thanks to authentication and encryption mechanisms [4], for military applications, civilian GPS variants, which do not have protection mechanisms, are frequently used. Consequently, civilian GPS is highly vulnerable to spoofing attacks [3].

A GPS spoofing attack entails consequences associated with the fall (crash) of the UAV or its interception and redirection along a different flight path. In both cases, these problems are related to cyber security. A targeted attack that takes control of a UAV or destroys it can harm everyone in the UAV’s flight area or damage other vehicles [5]. One of the main security concerns of UAVs is GPS spoofing attacks [6]. The civil GPS specification is publicly available [7], which makes the signal highly predictable and increases the potential risk of tampering. Therefore, GPS spoofing has become an important research topic since an attacker could hijack a UAV, use it to eavesdrop, or attack people or objects remotely.

Currently, many different methods of detecting GPS spoofing attacks are known [8,9,10,11,12]. Some of these methods allow the UAV’s GPS receiver to detect spoofing attacks [8]. These techniques include the ability of the GPS receiver to analyze the received signal strength and to compare it to the normal signal strength over time. UAVs can monitor GPS satellite identification codes or continually check the time intervals to ensure their constancy. These methods can be efficient in detecting attackers with weak capabilities. However, such methods may be ineffective against complex attacks, in which an attacker can falsify the coordinates of the victim with great accuracy [9]. O’Hanlon et al. proposed a method for detecting GPS spoofing attacks based on the use of two GPS receivers to check their cross-correlation [10]. This method has been tested for multiple attack scenarios and the authors have proven that it successfully detects attacks, although the method is unable to distinguish spoofing signals from genuine GPS signals and cannot detect spoofing when the signals are weak [13]. Other methods to prevent GPS spoofing, such as autonomous monitoring of receiver integrity, signal-to-noise ratio measurements, and Doppler shift detection, have been discussed [6,14]. For instance, Shepard et al. proposed a method allowing the UAV to detect the source of GPS spoofing using a ground station that continuously analyzes the content and time of arrival of information about the estimated location of the UAV [2]. It has been shown that their method is efficient and detects spoofing attacks in less than 2 s and determines a counterfeit’s source location after monitoring for 15 up to 150 m. Solove uses the automatic GPS signal gain control in a GPS receiver to detect and alert potential spoofing attacks within the method [15].

The ability to use multiple receivers to detect GPS spoofing attacks has been presented in several previous works [16,17,18]. Jansen et al. [17] used several independent GPS receivers to detect GPS spoofing attacks. Their proposed method depends on the distance between the receivers and then measuring the distance between the specified locations of the receivers. With the same GPS signals, the measured distances are the same as the previously recorded distances. However, in a GPS spoofing attack, the distances obtained will be very close to zero, since all receivers transmit information where the same location is indicated. Montgomery et al. demonstrated the possibility of using a receiver with two antennas for detecting GPS spoofing attacks [16]. Their proposed method is based on fixing the difference in carrier frequencies between different antennas tied to the same generator. In their configuration, the attacker must use an additional transmit antenna for each additional receiver antenna, which complicates the attacker’s task. Heng et al. [18] use multiple receivers to confirm the authenticity of GPS signals based on correlation with the military GPS signal without the need to decode it. This uses cross-validation receivers to authenticate the GPS signals. It has been shown that the proposed technique is efficient even when cross validation receivers are tampered with. This method has been tested on fixed and moving GPS receivers and shown to be effective in detecting spoofing attacks.

Panice et al. [19] presented an approach to detecting a GPS spoofing attack for a UAV, based on state analysis using a Support Vector Machine (SVM), which is used as an anomaly detection tool. They present solutions for detecting anomalies and a simulation environment for GPS attacks to assess the functionality and performance of the method. Their approach does not require additional equipment, and so it can be implemented on a small UAV. On the other hand, if the intruder has absolute knowledge of the positioning and trajectory of the UAV, he can go unnoticed by the system, causing significant false operations. Nonetheless, since usually the intruder does not know the actual trajectory of the UAV, the risk of a false alarm is small. Thus, the system can detect any spoofing attack.

Eldosouky et al. [20] suggested a protective mechanism based on the concept of co-localization [21]. Their mechanism is a methodology that allows a UAV to determine its real position in a two-dimensional coordinate system using the location of three other UAVs. It is assumed that each UAV has a device for measuring the relative distances to other neighboring UAVs. The co-localization of the UAV selects any three adjacent UAV to update their location, assuming that the selected UAVs do not lie on a straight line. The UAV can then pinpoint its 2D position accurately. With a covert GPS spoofing attack, the UAV cannot trust its GPS location or the location of other UAVs. However, the choice of a nearby UAV for the co-localization mechanism is fraught with risk since the UAV itself may be compromised. To overcome this limitation, the authors propose a protection mechanism based on the assumption that an attacker using GPS spoofing can attack only one UAV [20]. In their proposed mechanism, the UAV will use the location of four adjacent UAVs, instead of three, to determine its real location by identifying the UAV under attack and excluding it from calculations. This mechanism has the same cooperative localization requirements (i.e., the UAVs are non-collinear), UAVs can query the location of other UAVs through communication between them, and each UAV must be able to measure distances to neighboring UAVs. Thus, it should be noted that their method has many restrictions on its applicability.

Qiao et al. presented a method of counteracting GPS attacks based on the use of a technical vision system, which additionally allows calculation of the UAV’s speed and some other indicators and correlates them with the data received from the GPS [22]. Thus, the main methods of protecting UAVs from attacks related to GPS signal spoofing are based on the analysis of the state change in comparison with the reference of one sensor or on the correlation of data obtained from several GPS sensors. In some methods, in addition to GPS, other sensory mechanisms such as an accelerometer magnetometer and others are used to improve the quality of flight [23]. In this case, the correlation can take place both between devices of one UAV and between devices of a group of UAVs [24].

Semanjski et al. presented a method for determining the authenticity of a GPS signal based on the correlation between true and incoming signals using C-Support Vector Machine (C-SVM) classification [25]. Application of their method is suggested at the signal receiver level to avoid changes that may occur during signal processing. To train their classifier the authors used both data obtained under laboratory conditions and in real cases of attacks. They believe that the supervised machine learning-based approach (C-SVM) has great prospects since they managed to achieve positive results. The model they presented has achieved high detection rates compared to previously existing ones. In addition, by including a real meaconing event, the complexity of the model was improved. Thus, supplementing training datasets with real-world scenarios appears to be a valuable contribution to safety-critical models such as the detection of attempts to manipulate Global Navigation Satellite System (GNSS) signals. However, a disadvantage of this approach is the significant time and effort required for preparation and elaboration of the data set for training.

Kwon and Shim proposed a method for direct detection of GPS spoofing attacks using a positioning system based on an accelerometer [26]. They conducted a performance analysis using probability density functions. The difference between the measurements obtained by the accelerometer and a GPS receiver is used to detect an attack. The value of the error is used to obtain decision variables indicating the presence of an attack. The performance of two decision variables was compared by calculating the spoofing detection probability and the detectable minimum spoofing acceleration, considering the predetermined false alarm probability and the predetermined detection probability. The decision variables represent the change in the horizontal and vertical acceleration, both of which must be used together. Then the authors add another variable for the solution of the northern acceleration. The GPS spoofing detection method suggested in their paper is thus influenced by the acceleration error. Flying or driving conditions can affect GPS spoofing detection performance. For instance, if a ground vehicle encounters obstacles in the road, such as potholes, bumps, etc., its accelerometers can show large changes and degrade the ability to detect GPS spoofing.

Guo et al. presented a method for detecting and countering spoofing [27]. They used an algorithm based on Maximum Likelihood (ML) to solve the problem of multipath reduction to anti-spoofing. The authors presented tracking channels using multi-correlators and proposed a set of actions to detect and remove fake signals, to ensure that the receiver blocks the signals during a spoofing attack. Due to the complexity and variety of interference implemented by an attacker, using one anti-spoofing method may not prevent all types of spoofing. For example, an anti-spoofing method based on a multi-correlator structure can help block the signal. However, if spoofing started during data collection, this method will not be able to distinguish the genuine and fake recipient signals. Experimental results show that their proposed anti-spoofing method allows estimating the time delay, amplitude, and phase of the carrier of a fake signal with a small delay, and then eliminating it by adding the opposite fake signal. In addition, this method can perform a self-test, so that it can directly distinguish the phase of the genuine signal when the fake signal has a long delay. Finally, their proposed method can ensure that the receiver keeps the authenticated signal blocked when the correlation peak of the fake signal overlaps or moves away from the correlation peak of the authenticated signal and can further ensure that the receiver is positioned correctly in the presence of spoofing.

However, many authors say these previous methods are prone to false positives. Instead we present a method for detecting anomalies and attacks on UAVs based on the analysis of data collected and analyzed from the UAV’s own sensors only. The analysis compares normalized readings of the sensors at different points in time and identifies the degree of difference between them.

This offers a new method for detecting anomalies and attacks on UAVs based on the analysis of changes in the UAV’s state. The main difference of our method from the methods discussed above is that, to detect an attack, we do not need reference values or need to correlate the collected data with additional GPS receivers or other external systems. Our “self-diagnosis” method is based on the UAV’s ability to detect critical changes in its own behavior over time. The analysis is based on the collection and processing of data received from various parts of the UAV’s sensor system. Any sensors that produce numerical readings can be used. At the same time, as experimentally confirmed, our method makes it possible to detect accurately the presence of anomalous behavior and identify an attack.

The main purpose of this article is to present our method for detecting UAV sensor anomalies under the influence of a GPS attack. Therefore, the following tasks should be solved:

- To develop and implement a stand for carrying out an attack on a UAV;
- To carry out flight tests with a UAV in normal mode and during an attack;
- To collect the results in the form of UAV logs;
- To analyze the logs and identify the parameters that are susceptible to attack;
- To get raw data for testing the method;
- To develop a method for detecting anomalies for a UAV and to prove its effectiveness.

## 2. Materials and Methods

As a result of theoretical and experimental analysis, we identified a set of the most informative parameters that may indicate a cyber attack. These parameters are: CPU workload, UAV flight altitude (*h_a_*), satellite fix status (*G_n_*), GPS uncertainty (*G_u_*), and GPS noise (*G_noi_*) when detecting a GPS spoofing attack. To analyze changes in these parameters, it is necessary to choose an appropriate type of probability distribution. The choice is based on the fact that these parameters are discrete. Let us consider the most common distribution laws and assess whether they are suitable for the given parameters presented above or not. If the number of occurrences of a random event per unit of time is available, when the fact of the occurrence of this event in each experiment does not depend on how many times and at what points in time it happened in the past, and does not affect the future, and tests are carried out under stationary conditions, then Poisson’s law is usually used to describe such a quantity. Poisson’s law is also called the law of rare events. Thus we used the Poisson distribution law because, when analyzing the parameters in this study, it is necessary to assess the occurrence of unexpected peak values, which are rare events during the total number of operations of the UAV [28]. The Poisson model *P*() is typically used to describe a rare events scheme [29]. Under some assumptions on the nature of random events (the changes observed are random, independent, and discrete events), their number occurring over a specified time interval or a space area often obeys the Poisson distribution:

(1) $$P(K_{n})=\frac{\lambda^{K_{n}}}{K_{n}!}e^{-\lambda },$$

(2) $$P(Gn)=\frac{\lambda^{Gn}}{Gn!}e^{-\lambda },$$

(3) $$P(h_{a})=\frac{\lambda^{h_{a}}}{h_{a}!}e^{-\lambda },$$

(4) $$P(G_{u})=\frac{\lambda^{G_{u}}}{G_{u}!}e^{-\lambda },$$

(5) $$P(G_{noi})=\frac{\lambda^{G_{noi}}}{G_{noi}!}e^{-\lambda },$$

where *P* is the probability function of the distribution of a random variable according to Poisson’s law; $K_{n}$ is the percentage of the total amount of processor time in the interval between *n* and *n* − 1 that the processor spent on processing tasks when running in the kernel mode; *h_a_* is the UAV’s flight altitude; *G_n_* is the state of fixation by satellites (i.e., the number of satellites from which the UAV receives position information); *G_u_* is the GPS’s uncertainty; *G_noi_* is the GPS’s noise (these parameters are discrete); λ is the mathematical expectation (the average number of events of interest per unit of time); and *e* is Euler’s number.

To obtain the distribution, ten random variables are used. Then the sliding window concept is used to update the distribution information. Six previous values and four new ones are taken and a new distribution is constructed. This concept allows fixing a sharp increase in values. Further, to determine the presence of an anomaly or a state change, it is necessary to determine to what extent the distributions differ from each other. Comparison of distributions rather than raw data gives a more accurate result. Raw data can vary dramatically. At the same time, the changes and observations obtained based on raw data will not indicate an anomaly or attack. Normalizing the raw data and bringing it to a suitable distribution will capture the change in the system. If the entropy values are used (i.e., differences between distributions), an anomaly can be detected. The Kullback-Leibler divergence can be used to calculate the entropy value, for example as in Afgani et al.’s work [30]. In this case, it is necessary to use not an integral value, but a sum, since we are talking about discrete quantities:

(6) $$D(P_{n}(\lambda_{n})\Delta t||P_{n}(\lambda_{n})(\Delta t-1))=\sum_{n_{i}\in N}P_{n}(\lambda_{n})\Delta t*\ln\frac{P_{n}(\lambda_{n})\Delta t}{P_{n}(\lambda_{n})(\Delta t-1)},$$

where $P_{n}(\lambda_{n})\Delta t$ is the Poisson distribution for a given exponent *n* for the current period Δt; $P_{n}(\lambda_{n})(\Delta t-1)$ is the Poisson distribution for a given exponent *n* for the previous period of time; and ln is the natural logarithm.

Then, our algorithm for detecting anomalies is as follows:

1. Fixation of the raw values of the analyzed parameters for a certain period of time.
2. Plotting a suitable distribution type for the collected parameters.
3. Selection of the previous values and supplementing them with those collected at a new point in time, to build a time series of values using a sliding window.
4. Construction of a new distribution for new values according to the same distribution law.
5. Calculation of the Kullback-Leibler divergence between the two distributions.
6. The higher the obtained value of the Kullback-Leibler divergence, the more likely it is that the system has been influenced in the form of an attack or external destructive influence. Typically, this value should be greater than or equal to two. This value was chosen with the condition that the divergence should tend to zero and not exceed one. But to avoid unnecessary false alarms, the threshold was increased to two, which was confirmed experimentally.
7. Repeat the algorithm for subsequent values, starting from step 3.

**Figure 1:**
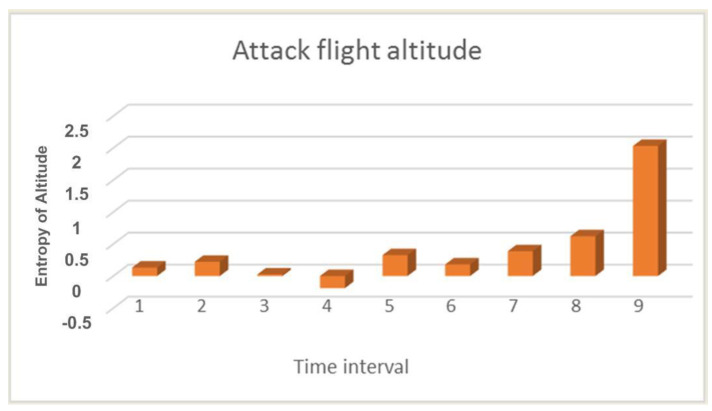
The result of calculating the Kullback-Leibler divergence of the deviation of the flight altitude indicator during an attack.

Therefore, the higher the entropy, the more likely it is that abnormal behavior is observed. Abnormal behavior can occur not only due to the attack but also due to environmental conditions. Thus, for example, changes in the engine speed and flight altitude may not be related to an attack. To unambiguously identify an attack, it is necessary to analyze several indicators at once and determine the degree of their deviation. Figure 1 shows the calculation of the entropy value for the UAV flight altitude during an attack.

**Figure 2:**
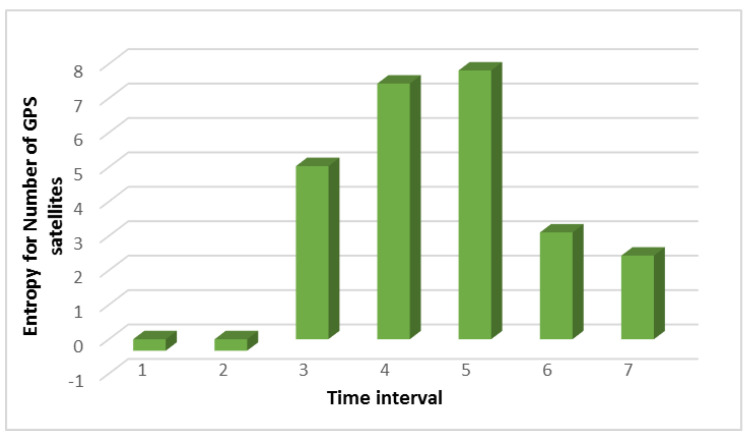
The result of calculating the Kullback-Leibler divergence for the indicator “number of GPS satellites” at the time of the attack.

Since the attack on the UAV did not begin immediately, the first few values in Figure 1 are quite low, and so we can see an increase in value only in the last three calculations. This is because the UAV under attack may crash and has already begun to descend sharply, but as a result of operator intervention in the crash process, it was prevented. But as mentioned earlier, a lowering of the flight altitude can only indirectly confirm an attack. The most indicative parameter is the number of GPS satellites. The parameter for this calculation is shown in Figure 2.

**Figure 3:**
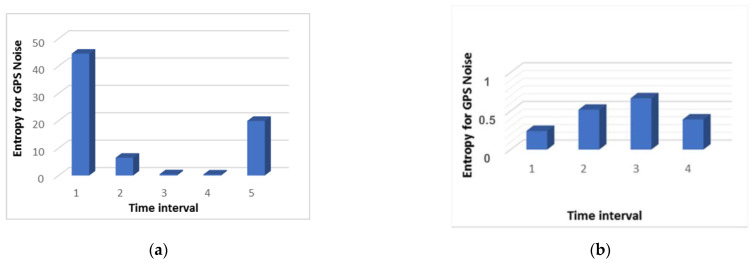
The result of measuring the Kullback-Leibler divergence of the GPS noise index: (**a**) During the attack; and (**b**) under normal conditions.

Figure 2 shows that high values begin in interval 3. This does not mean that the number of satellites has increased as it also could have decreased. The main thing is that the type of distribution between intervals 2 and 3 does not coincide, and this indicates that significant changes have occurred when the number of satellites should be approximately the same. It should be noted that the comparison did not take place with the data obtained in a normal situation. Namely, the data obtained from the different time intervals in the same overall period of the UAV’s flight was compared. For example, let us compare the results of searching for anomalies when analyzing the noise level with and without an attack, as shown in Figure 3.

Figure 3 shows that during the attack, an increase in the entropy value is observed three times. In other words, we can say that it took place throughout the entire flight. During the normal flight, the entropy value remained at the minimum level. This does not mean that there were no deviations during normal flight. They were simply insignificant and did not affect the quality of the flight.

## 3. Results and Discussion

For the experimental study of our method, a quadcopter UAV whose architecture was developed for testing attacks and analyzing security threats was used. Table 1 presents the main characteristics of the experimental stand.

One of the scenarios is that the quadcopter, having determined its current position, receives the static coordinates of its target. A copter-type UAV moves to a given point and fixes its position in space, while maintaining its altitude. In the case of an external physical impact, for example, a natural factor or impact from another object, the UAV’s autopilot system increases the engine speed and sets it in the opposite direction to maintain a given position. When the UAV is displaced from its preset position, the position hold system increases the engine’s RPM power depending on the distance between the preset point and the actual location of the UAV. When the UAV returns to a given point, the autopilot enters the normal altitude control mode. In the scenario of maintaining a position with fixed coordinates, an attacker could influence the direction and speed of movement. After the start of the attack, the quadcopter navigation system perceives the broadcast signal as real, thus the actual location of the UAV differs from that determined by the navigation system. After the autopilot system detects the displacement, the UAV starts moving in the opposite direction. Thus, by indicating a fake offset on the circle, where the center will be the fixation point of the UAV, you can set the direction of movement. The attacker’s task is to smoothly shift the fake geo position from the center, that is, the place where the quadcopter is fixed, to the point of the circle. By adjusting the radius of this circle, the attacker can influence the speed of the quadcopter. By adjusting the speed and motion vector, the attacker can control the location of the UAV. An important factor is that when broadcasting a fake position, the attacker must independently consider the physical impact on the UAV. To counteract external factors, it is important to determine the real position of the quadcopter.

**Figure 4:**
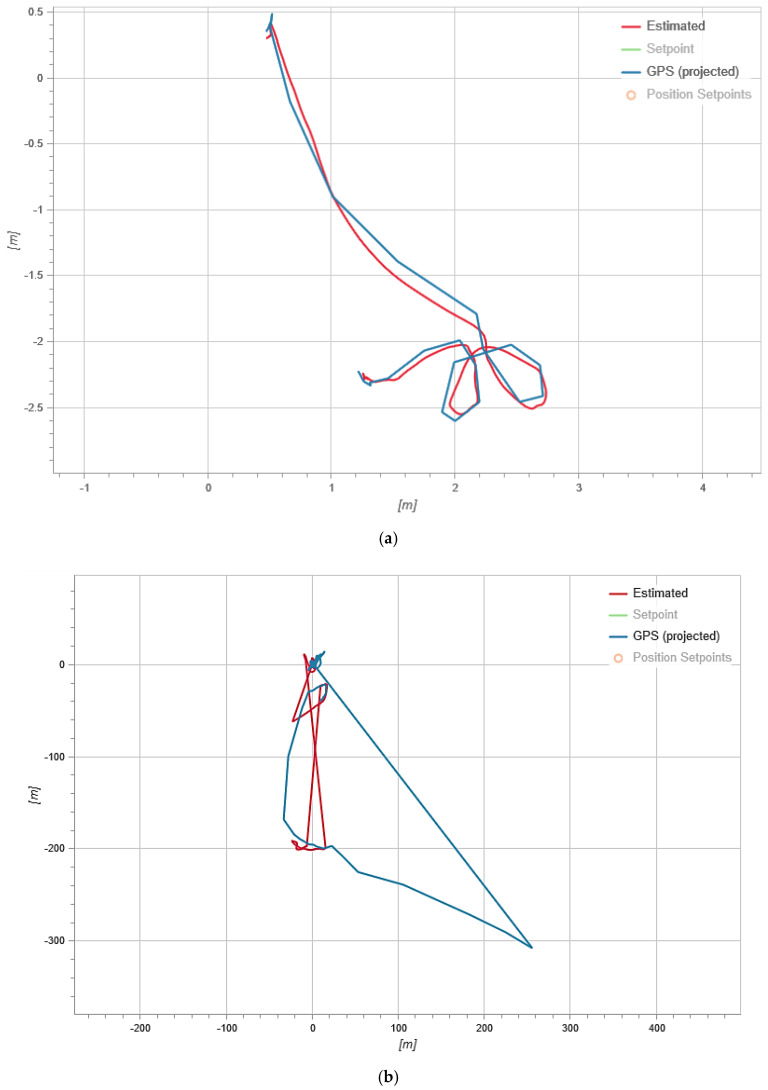
UAV flight trajectories: (**a**) In the normal mode; and (**b**) during a GPS attack.

During our experimental study, an attack was carried out on the UAV five times, while the positive effect took place every time. Let us consider the data collected from the logbook during the attack and normal UAV flight. Figure 4 shows the result of fixing the UAV’s flight trajectory.

In Figure 4, the red line shows the estimated trajectory (Estimated) which is the flight path calculated by the on-board computer according to the accelerometer. The blue line shows the GPS trajectory (GPS projected) which is a trajectory built based on GPS module data.

As we can see from Figure 4a, both trajectories coincide during the normal flight and this indicates the normal operation of the UAV. Figure 4b shows that during an attack, the GPS trajectory differs from the UAV trajectory recorded by the internal sensor. Since the attack was aimed at spoofing GPS data, it led to the difference in trajectories, because many GPS systems are only updated once a second (and high-performance GNSS devices are updated five to 10 times a second) and often have an accuracy of ±1 m only in good conditions. The condition evaluator uses other sensors to fill in the gaps between 1 s GPS updates and thus improves accuracy.

**Figure 5:**
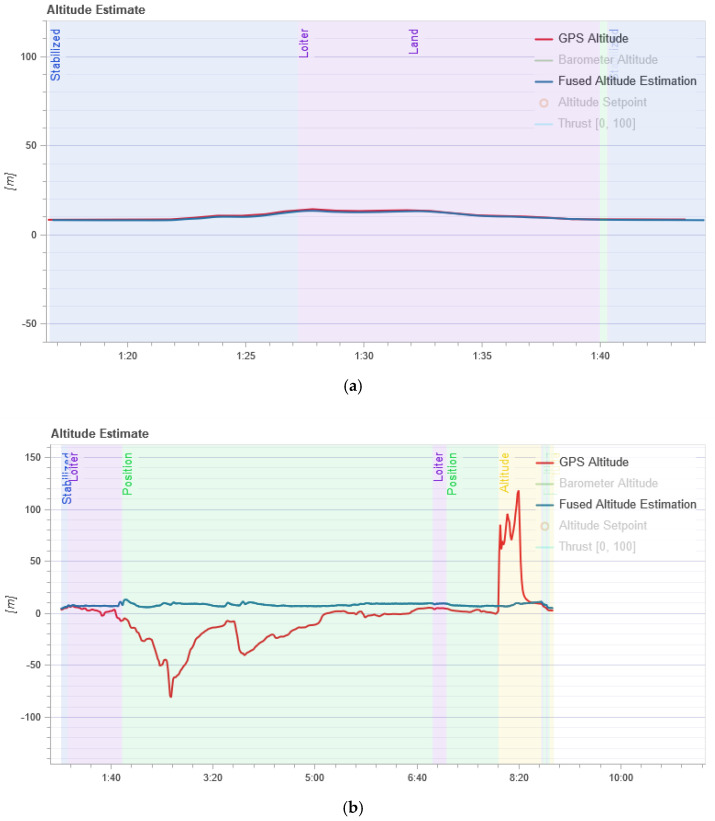
Readings of flight altitude sensors: (**a**) In the normal mode; and (**b**) during a GPS attack.

Next, it is necessary to analyze the UAV’s flight altitude in the normal mode and during an attack since this parameter affects the detection of an attack. Figure 5 shows the result of changing the UAV’s flight altitude in the normal mode and during an attack.

In Figure 5, the red line (GPS Altitude) is the UAV flight altitude according to the GPS module, and the blue line (Fused Altitude Estimation) is the estimate of the change in altitude. Fused Altitude Estimation is the result of the state estimator, and so it uses not only the raw GPS data but also the accelerometer. As shown in Figure 5a, the GPS altitude and the final altitude coincide during normal UAV flight. Figure 5b shows that the altitude changes and the GPS altitude graph and the final altitude graph do not coincide during an attack on the GPS.

**Figure 6:**
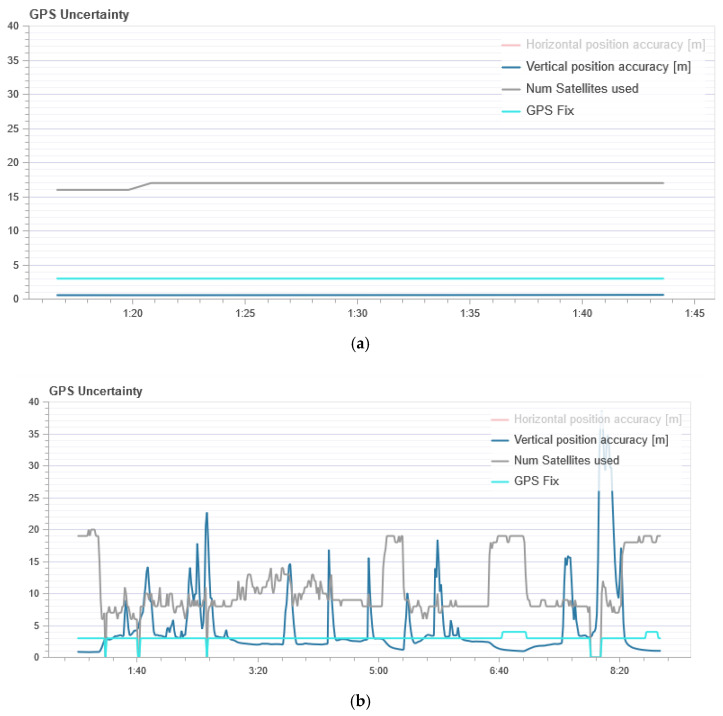
The state of the parameter indicating the number of GPS satellites: (**a**) In normal UAV flight mode; and (**b**) during a GPS attack.

Next, we analyzed the data on the number of satellites that the UAV captures during regular flight and under the influence of an attack, as shown in Figure 6. In Figure 6, the gray line (Num Satellites used) is the number of satellites used. The blue line (GPS Fix) is the state of fixation by satellites. The GPS uncertainty graph shows information from the GPS device. The number of satellites used must be about 12 or higher.

Figure 6 shows that the GPS fix (blue line) is constant during normal flight. It means that the drone does not lose communication with satellites and always knows where it is. The average number of satellites used is 18 (gray line). During the attack (Figure 6), communication with satellites is not stable and the number of satellites is constantly changing. You can also see when the UAV loses its GPS fix, the number of satellites used to determine the location is 0.

**Figure 7:**
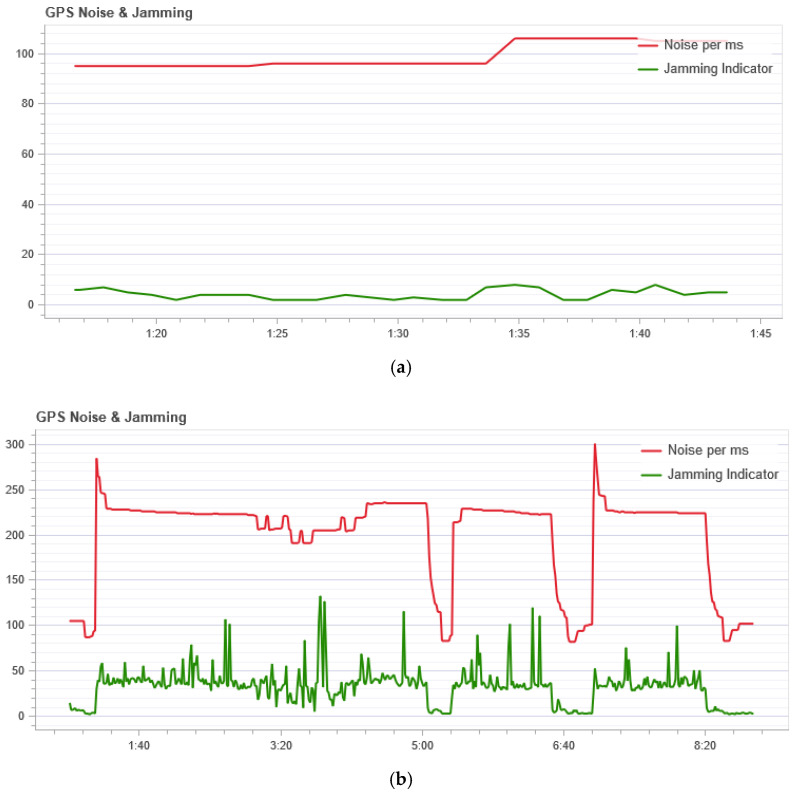
Indication of the level of noise affecting GPS: (**a**) In the normal mode; and (**b**) during a GPS attack.

The GPS noise sensor readings are shown in Figure 7. In Figure 7, the red line (Noise per msec) is the GPS noise. The GPS noise and interference graph is useful for checking for GPS signal interference. The GPS signal is very weak and; therefore, can be easily disturbed/jammed by components transmitting (via cable) or transmitting at the frequency used by the GPS. The interference indicator should be around or below 40. Values around 80 or above are too high. Signal interference results in reduced accuracy and less satellite usage. Figure 7 shows that during normal flight the noise does not exceed 105 per ms, but during an attack, this indicator is not stable and at the peak has a value of 300 per ms. So we can conclude that during the attack a high noise level is in the GPS channel.

**Figure 8:**
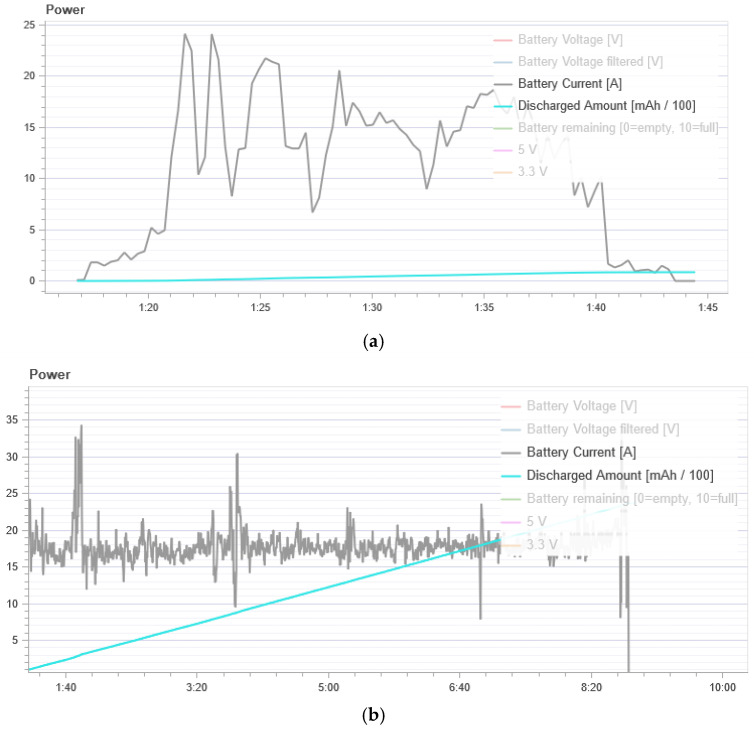
Energy consumption curves: (**a**) In the normal mode; and (**b**) during a GPS attack.

Figure 8 shows energy consumption. In the normal flight mode, the UAV has an average power consumption of 500 to 1000 mAh. During the attack, we see a strong power consumption of 10,000 to 40,000 mAh. Thus, we can conclude that the UAV spends much more energy to maintain its flight when it is under attack.

**Figure 9:**
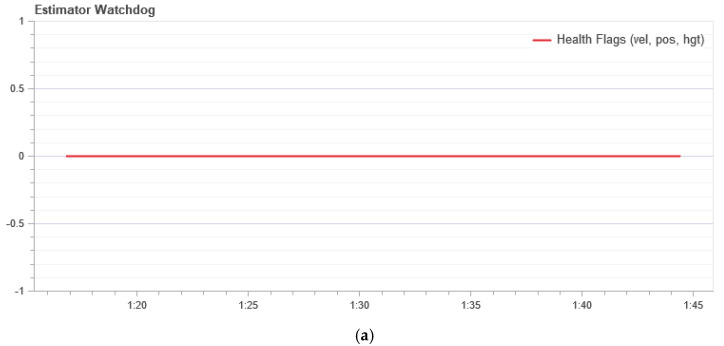

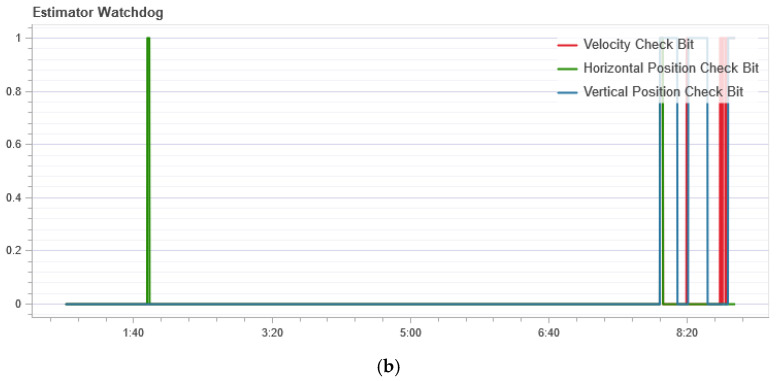
The state of the system performance evaluator: (**a**) In the normal mode; and (**b**) during a GPS attack.

Figure 9 shows the measurement result of the condition evaluator. It should be a constant zero. If one of the flags is nonzero, the evaluator has encountered a problem that requires further investigation. In most cases, this is a problem with the sensor, for example, magnetometer interference.

**Figure 10:**
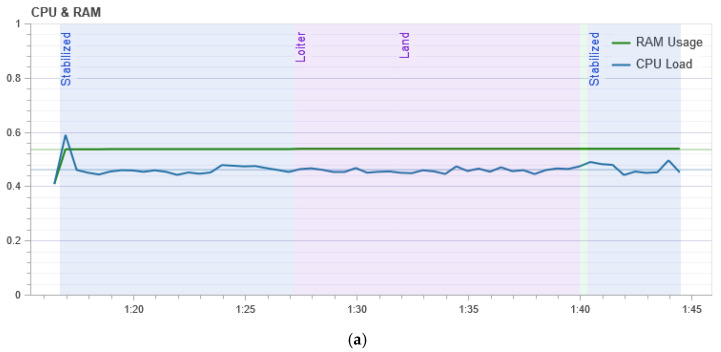

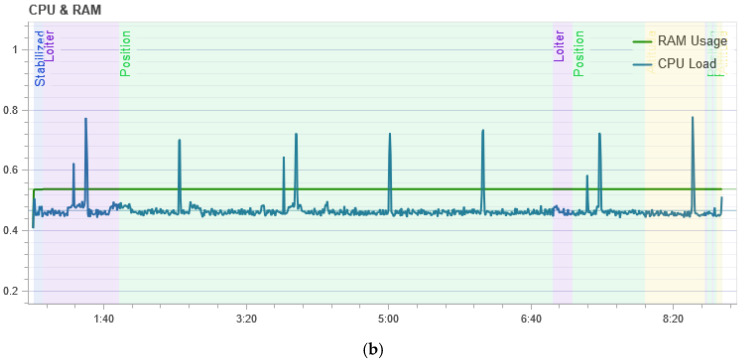
Loading the UAV processor: (**a**) In the normal mode; and (**b**) during a GPS attack.

Figure 10 shows the result of measuring the processor load. It shows that in the normal mode of UAV flight, the central processor of the flight controller is loaded at its peak by 45%, and during the attack, the load is not stable and reaches 80% at its peak, which means that the drone requires more computation during the attack to maintain its flight stability.

From Figure 5b we can see that the flight altitude is rather uneven during the attack, especially in the last time intervals when there is a sharp rise. This change in altitude is correlated with the calculation of entropy which is shown in Figure 1. The figure shows an increase in the entropy for the flight altitude indicator, which indicates the presence of an anomaly. The parameter number of GPS satellites constantly changes during an attack, and this is clearly seen from Figure 6b. If there is no attack, this graph looks like a straight line. At the same time, it can be seen from Figure 2 that in the second time interval the level of divergence has already grown significantly. This indicates the presence of serious changes in the system and may indicate an anomaly. At the same time, we draw attention to the fact that the numbers with a change in the altitude and the number of satellites have different orders, but for our method, this turns out to be not important. Due to the use of the probability distribution, we can recognize that the value of the divergence has increased by more than two and this is enough to detect the anomaly.

Unlike previous work [23,24,25,26], we do not need to know which behavior is considered normal, or to compose many training datasets. It is enough for the UAV system to obtain one value and it does not need to be compared with a normal value, as in other approaches [8,9,10,11,12]. Our solution compares observable variables only using their own value in the previous and current periods of time.

From Figure 7a, which shows the result of measuring the GPS noise level, it can be seen that even during normal operation the graph is not a straight line and some noise is evident with changes in its level. At the same time, as shown in Figure 3b, the Kullback-Leibler divergence is at a low level, that is, the system does not consider this behavior an anomaly. This is due to several factors. Firstly, the change in the noise level with normal behavior is not significant. Secondly, due to the algorithm for processing incoming data, which is presented in Section 2, during data processing, the series are constructed in such a way that the probability distribution does not change its form too much with small changes.

This cannot be said about the situation with the GPS noise during an attack. In this situation it changes significantly, as can be seen from Figure 7b. As a result, the divergence value during the attack is quite high immediately after the first interval, as can be seen in Figure 3a.

Even though the attack is directed at the GPS system, it ultimately affects several parameters at once. These are both the flight altitude and GPS noise and the number of GPS satellites and the level of CPU utilization and energy consumption. For other attacks, such as the eavesdropping attack presented by Wang et al. [31], other sets of parameters can be analyzed and applied. An attack can be detected precisely by a combination of factors. In future papers, a decision-making system will be presented to determine both the attack and, by analyzing several parameters and the degree of their changes, the type of attack. It is difficult to do this using just one parameter, since weather conditions and noise attacks, etc. can also be the cause of changes. In this paper, we focused on GPS spoofing attacks only and studied in detail what parameters are affected and to what extent.

Thus, our presented method, based on the analysis of changes in the internal state of the UAV, makes it possible to accurately detect the presence of anomalous behavior and diagnose a possible spoofing attack. The novelty and advantages of our method are as follows.

- It is versatile and can be applied to various data sets obtainable from the particular UAV’s sensor system.
- It determines only that a change has occurred in the system and does so quite accurately; it is then up to the decision-making system to determine whether the change is an attack or not.
- It does not require information about reference and normal values, or inputs from external sources. The UAV independently analyzes the changes in indicators and compares its own state at different intervals. If the state is stable or quickly becomes stable, it means that there are no anomalies.

## 4. Conclusions

Although a large number of methods have been published for countering UAV navigation system spoofing attacks, this topic is still relevant. To date, a number of successful attacks on the navigation system of UAVs have been demonstrated. Our new self-diagnosis method for analyzing and detecting system anomalies has several advantages that distinguish it from other methods.

First of all, the method is universal and can be used for any UAV subsystems from which numerical readings can be taken. The main thing is to determine the correct type of probability distribution for the analyzed parameter. Secondly, the method is lightweight and energy efficient. The software implementation of the method has little impact on CPU load and the UAV’s energy consumption. Thirdly, since the method allows analyzing any parameters, it does not matter what configuration the UAV has, and so the approach can work with whatever data is available. Fourthly, using our method, it is possible not only to detect anomalies but also to determine a change in the patterns of UAV behavior and a change in its states. If the values of entropy are not too high, and a single increase takes place, then this may indicate a change in flight mode. Correlation of the analyzed parameters can unambiguously reveal an attack and determine its type. Each attack affects a certain number of subsystems so the type of attack can be characterized by which parameters are affected. The data collected in the form of time series can be used in the future to train a neural network, which can be trained on these sets and help to decide about the presence of an attack.

This method can be implemented and applied to detect attacks on the GPS system installed on a UAV to ensure its safe navigation. The method can be integrated into any type of UAV. In addition, the method can be used for the analysis of other sets of parameters and applied not only to UAVs, but to any cyber-physical system.

## Figures and Tables

**Table 1:** Experimental stand characteristics.

Part Name	Model
Flight controller	Pix Hawk 4 (STable 10.1 firmware)
Frame	S500
Speed controllers	XT-XINTE 30A
Telemetry	3DR Radio Telemetry 915 MHz 100 mW Aerial Ground Data Transmission Module for Pixhawk 4
Receiver	FS-I6B
Battery	ZOP Power 3S 11.1V 4200 mAh 40C Lipo Battery XT60 Plug

## Data Availability

Data sharing not applicable.
